# Considerations on efforts needed to improve our understanding of the genetics of obesity

**DOI:** 10.1038/s41366-024-01528-0

**Published:** 2024-06-07

**Authors:** Sujoy Ghosh, Claude Bouchard

**Affiliations:** Pennington Biomedical Research Center, Baton Rouge, LA 70808 USA

**Keywords:** Genetics, Physiology

Investigations into the role of inheritance on the risk of obesity began about 100 years ago primarily with the work of CB Davenport [1]. He studied 528 parental mating and their 926 male and 745 female offspring, classifying subjects into five classes of BMI in an attempt to track Mendelian segregation from the parental to the filial generation. He reported a parental influence on the BMI of offspring, but all mating types produced a variable progeny in terms of BMI classes. His research generated suggestive evidence, entirely compatible with current models, to the effect that body weight regulation is complex and multifactorial, with some degree of inheritance. In the last 50 years, research on the genetics of obesity has enjoyed growing attention buttressed by major advances in genomics, study designs, analytical tools, and high throughput technologies.

Where we stand on the genetics of obesity and related phenotypes has been recently reviewed [2]. We will not dwell on this issue herein. Rather, in this Perspectives paper, we briefly comment on topic areas deserving attention because of their potential to enhance the quality and power of future studies on the genetics of obesity.

## Targeting the most relevant phenotypes

Most genetic research has concentrated on body mass index (BMI), yielding significant insights. As recently reviewed, criticisms of BMI emphasize its limited capability in accurately reflecting body adiposity levels, fat distribution, and the health risks associated with obesity [3]. From a population perspective, we think this view is overly pessimistic. BMI is highly correlated with total body fat or body fat percentage, with correlation coefficients of ~0.90 [4, 5], in agreement with extensive literature.

Although BMI does not provide direct information about fat distribution, it correlates significantly with waist circumference (*r* > 0.9) and abdominal visceral fat. The critique that BMI inadequately represents obesity’s health risks is also not fully supported by data. Multiple cross-sectional and prospective epidemiological studies have linked BMI with mortality, cardiovascular diseases, type 2 diabetes, hypertension, certain cancers, osteoarthritis, and other conditions. Excess weight is a clear health risk, escalating with BMI level.

Given this, genetic studies focusing on BMI and its fluctuations over time or due to interventions are invaluable, especially when examining average or group effects. However, since genetic research often aims at discovering genome-driven individual differences, precise phenotyping is also necessary to enhance success in genetic investigations. Although BMI correlates with multiple obesity phenotypes, 10 to 20% or more of population-level variance remains unexplained by BMI (*r*^2^ × 100). Moreover, the standard error of the prediction estimate is rather large when adiposity or energy balance endophenotypes are predicted from BMI. Additional advancements are expected from studies focusing on precise phenotyping of total adiposity, lean mass, adipose tissue distribution, ectopic fat depots, resting and sleeping metabolic rates, thermogenic response to food, exercise adaptation, and dietary and physical activity behaviors, despite their likely smaller sample sizes. This is exemplified in a recent report on the association of common variants with MRI-derived, BMI-independent measures of distributed adiposity [6].

## Opposing forces of obesity alleles vs. thinness alleles

The prevalence of obesity and thinness demonstrates significant familial aggregation, with heritability levels for lean mass and thinness comparable to those for overweight and obesity. In a study contrasting 1456 individuals with severe, early-onset obesity with 1471 healthy, persistently thin adults (mean BMI: 17.6), a panel of ~1.2 million genotyped and imputed markers accounted for 32% of the liability for severe obesity and 28% for persistent thinness [7]. While less frequent than those on obesity, genome-wide association studies (GWAS) have identified multiple loci linked to thinness [7–11]. Additionally, the availability of large sample sizes opens avenues to explore the effect size distribution of obesity or leanness alleles across the BMI or adiposity spectrum. Early evidence indicates that obesity alleles have substantially larger effect sizes in individuals with obesity compared to those who are overweight or of normal weight [2, 12]. A similar pattern could be hypothesized for leanness alleles, but in the opposite direction.

Several key insights have already emerged from these studies. First, comparing individuals with severe obesity to healthy thin subjects is an effective approach for identifying loci contributing to each or both conditions. Second, there are loci specifically associated with the predisposition to severe obesity, but not to thinness. Third, some loci are linked to persistent thinness in healthy individuals but not to obesity risk. Fourth, specific loci, such as FTO and MC4R, contain distinct alleles associated with severe obesity or thinness. Finally, integrating research on alleles for both thinness and obesity in large-scale studies could significantly enhance our understanding of obesity genomics. Recognizing and documenting obesity-opposing alleles could lead to a more precise and all-encompassing perspective on the genetics of obesity.

## Genomic architecture of the obesity genetic component

The first GWAS investigating obesity were reported in 2007 [13, 14]. These studies, along with genomic sequencing, have been pivotal in identifying alleles associated with obesity and related traits, especially as the study sample sizes expanded significantly [15, 16]. To date, over 60 GWAS have been conducted for obesity traits, revealing more than 1100 loci at the genome-wide significance level [16]. Importantly, multi-ethnic GWAS (e.g., in cohorts of Asian, Hispanic/Latino and African-American ancestries) have allowed for improved discovery and fine-mapping of genetic loci [17], such as the discovery of the obesity-promoting missense variant in the CREB3 regulatory factor gene (*CREBRF*) specifically in Samoans [18]. There is a need for increased representation of non-European populations for a comprehensive assessment of population-specific genetic architectures in obesity.

The total genetic component of obesity is unlikely to be accounted for unless all genomic constituents have been properly considered. This is a major task, requiring vast sample sizes in order to adequately cover the wide spectrum of common, low frequency, and rare variants. Rare variants are of particular significance as they may exhibit at times more than ten times the effect size observed with common alleles [19]. Additionally, exome coverage, often containing rare alleles, is vital. Importantly, most obesity-associated alleles reside outside gene coding sequences, necessitating a comprehensive approach that includes gene expression regulatory regions, introns, intronic junctions, potential methylation sites, sequences encoding non-coding RNAs, and copy number variants of DNA motifs of various sizes. Furthermore, investigations into gene-environment interactions, including age, sex, nutrient and caloric intake, energy expenditure, metabolic rates and physical activity levels, and the genomic foundations of assortative mating concerning obesity traits, are also crucial.

The primary limitation in contemporary obesity genetics research is sample size. However, substantial progress has been made in this regard. Since 2007, the average GWAS sample size has increased about tenfold [15]. Studies based on samples of one million or more subjects are now more common, enabling the identification of variants with very small effect sizes [15, 16]. We support the notion that larger BMI studies incorporating more in-depth genomic screening of common and rare variants can provide new insights into the causes of excess weight and adiposity [20], and could enable detailed comparisons of effect sizes across different weight, adiposity, or lean mass classes with robust statistical power.

## Non additive and nonlinear effects

Variations in BMI heritability estimates across populations and the incomplete explanation of heritability by genetic variants (“missing heritability”), suggest that gene-gene and gene-environment interactions might be additional mechanisms contributing to obesity. While there are reports of individual gene-nutrient (e.g., *APOA2*, *NPC1*) and gene-physical activity (e.g., *FTO*) interactions, the generally small effect sizes of obesity-associated variants hinder reliable estimation of these interactions suggesting that only variants with robust obesity associations are likely to yield meaningful results [21]. A comprehensive meta-analysis has reported significant interactions for a subset of BMI and waist-to-hip ratio (BMI-adjusted) associated SNPs with age and sex [22]. Future research, with sufficiently large sample sizes and precise trait selection, stands to gain from investigating obesity-related gene-environment interactions in each sex and across life stages [23, 24].

## Response heterogeneity in weight perturbations

Individuals vary in their responses to weight loss treatments, with some experiencing significant weight loss while others essentially maintain their initial body weight in response to the same treatment. The most convincing evidence for weight gain or weight loss heterogeneity in response to an intervention comes from controlled experimental overfeeding or negative energy balance studies [2, 25]. Research involving monozygotic twin pairs in such studies has shown pronounced within-pair similarity and between-pair variability, indicating that a substantial fraction of variability in weight gain or loss is under genetic control [26–28]. However, such experimental studies can only be undertaken with limited sample sizes and are not particularly useful for genome-wide explorations. Nevertheless, they are appropriate for the examination of the transcriptome, epigenome, proteome, and metabolome in relevant, accessible tissues, and can potentially lead to candidate DNA variants and genes for further exploration in these experimental contexts and larger-scale studies. Thus, experimental studies on overfeeding or negative energy balance could contribute significantly to identifying the proteo-genomic convergence in obesity research [29].

## The obesity epigenome

Epigenetic mechanisms, including DNA methylation, histone modifications, and non-coding RNA expression connect the environment to the genome, leading to supra-genomic adaptation. Several studies have identified genome-wide associations of DNA methylation with obesity and obesity-targeting interventions [30–33]. Certain genetic variations also function as methylation QTLs, influencing BMI through altered DNA methylation [34]. Notably, much of the adiposity-associated DNA methylation changes appear to be acquired early in life [33]. Histone modifications, such as acetylation and methylation, are also associated with BMI, both globally and at specific gene promoter levels [35, 36]. While these epigenetic changes are generally viewed as consequences, rather than causes, of excess adiposity [33, 37], opposing viewpoints have been proposed [38, 39]. Associations of non-coding RNAs, such as microRNAs and lincRNAs, with obesity have also been observed [40–42]. Most epigenetic studies are conducted on whole blood samples for practical, biomarker-focused reasons, but given the context-sensitive nature of epigenetic alterations, research in biologically more relevant tissues is necessary [43–45]. Future epigenetic research should additionally investigate proximal measures of adiposity and fat distribution, traits linked to food intake and behavior regulation, and experiments involving body weight perturbations.

## Our genome differs from the genome we were born with

The accumulation of mutations in the human genome has been a key driver of human evolutionary history, significantly influencing the genetic predisposition to obesity. To date, ~10 million common variants have been identified in the human genome [46], and over one billion predominantly rare genome-wide variants have been discovered [47–49]. An offspring inherits a set of haploid chromosomes and linked DNA variants from each parent. Additionally, new mutations occur in the zygote and throughout pregnancy, resulting in up to 100 novel mutations in a newborn that were absent in the parents’ germlines.

The genomes at conception, birth, maturity, and death are all slightly different from each other, resulting in increasing genomic variability among cells and tissues. Thus, somatic genomic mosaicism is a constant process, with new mutations accruing throughout an individual’s life. Most new variants are unique to a population of cells or to an individual, but some occur at mutation-prone sites and would tend to be more common. Some of these mutations can be beneficial but are more often neutral or increase disease risk. The NIH recently initiated a project to map these new variants across 15 tissues to assess their health impacts. Ultimately, this endeavor will necessitate longitudinal observations to identify new mutations and understand somatic mosaicism’s role in health and disease. Obesity geneticists stand to gain from incorporating these insights into their work, especially with the development of this new NIH resource.

## The promise of bioinformatics

High-content data from various molecular profiling platforms, including spatially-aware profiling, is poised to significantly influence obesity genetics research. Bioinformatics has already driven major molecular discoveries in obesity. Analyses focusing on pathways of GWAS variants linked to obesity have underscored the importance of both neuronal and peripheral mechanisms in regulating BMI and body fat [50, 51]. Integrative bioinformatics, combining GWAS signals, transcriptomic data, and regulatory genome profiles, has deepened our understanding of the genetic framework and potential therapeutic targets for obesity-associated traits [52, 53]. Moreover, bioinformatics has advanced our knowledge of obesogenic processes by identifying proteomic, metabolomic, and multi-omic markers signatures associated with obesity and adiposity traits [53, 54]. As genome sequencing becomes more prevalent, artificial intelligence and machine learning approaches for predicting variant function may become more feasible [55]. Explainable algorithms are already improving our understanding of obesity, such as through analyzing patterns in dietary interventions [56]. However, challenges persist in integrating and analyzing obesity-related data effectively. Decisions between union and intersection-based methods for integrated datasets, and the development of robust statistical approaches for gene prioritization are essential for enhancing bioinformatics analyses. Additionally, the creation and application of extensive “big” datasets, (e.g., the BigO project) [57], require advancements in statistical, machine learning, artificial intelligence, and database methodologies, enabling bioinformatics to address complex obesity-related questions more effectively.

## Biological correlations to genetic associations

In monogenic obesity, there is a generally clear link between genetic variants and biological functions, particularly for genes in the leptin and melanocortin signaling pathways (*LEPR, MC4R*, and *POMC*) [58]. The situation is more complex for polygenic obesity, where only a few GWAS loci (e.g., *FTO*, *TMEM18, CADM1/CADM2*, *NEGR1*) have been functionally followed up thus far. Assigning direct biological correlates to an allele is challenging due to several factors, including a lack of convergence with known obesity mechanisms, the need for comprehensive functional analyses, tissue-specificity of effects, divergent variant effects, and phenotypic heterogeneity [53]. One example is the widely replicated obesity-associated variants around the *FTO* gene, for which multiple mechanisms and multiple gene targets have been proposed, with no clear consensus [59–61]. Pathway and gene network-based analyses of obesity-associated SNPs show enrichment in lipid catabolism and oxidative phosphorylation pathways, and in CNS-driven processes. Knockout mouse phenotype-based interrogations suggest a focus on nervous system and weight-related phenotypes. However, large-scale BMI-association GWAS are often inconclusive regarding links between genetic variants and hormonal regulation, skeletal muscle metabolism, or energy expenditure pathways, underscoring the complexity and multifaceted nature of genetic influences in obesity [53]. This represents an area where further studies are clearly warranted.

## Conclusions

Obesity genetics sits at an exciting threshold between opportunities and obstacles. The availability of large genetic and multi-omics datasets and commensurate advances in bioinformatics and machine learning tools could allow for unraveling the molecular underpinnings of adiposity in unprecedented detail, leading to new insights into the origin, persistence, and treatment of obesity. However, generally small to very small variant effect sizes, biological redundancy, discrepancies between observed and genetically explicable heritability, and an over-reliance on population-level phenotypes (instead of more mechanistically aligned molecular and behavioral attributes) are some of the current challenges to obesity genetics. Genetic medicine shows significant promise in the field of monogenic obesity, especially with the approval of genotype-informed treatments for drugs such as metreleptin and setmelanotide [62, 63]. The situation in polygenic obesity is considerably more complex, although a recent exome sequencing study identifying obesity-protective variants in the *GPR75* gene is a promising discovery with therapeutic potential [64]. In addition to reliance on the numerous assets of modern genomics and genetics, integrative science incorporating relevant information from diverse domains is key to successful genetic obesity research, in recognition of the multifaceted nature of obesity.
